# Is high prevalence of Echinococcus multilocularis in wild and domestic animals associated with disease incidence in humans?

**DOI:** 10.3201/eid0703.010307

**Published:** 2001

**Authors:** B Gottstein, F Saucy, P Deplazes, J Reichen, G Demierre, A Busato, C Zuercher, P Pugin

**Affiliations:** University of Bern, Bern, Switzerland. bruno.gottstein@ipa.unibe.ch

## Abstract

We investigated a focus of highly endemic Echinococcus multilocularis infection to assess persistence of high endemicity in rural rodents, explore potential for parasite transmission to domestic carnivores, and assess (serologically) putative exposure versus infection frequency in inhabitants of the region. From spring 1993 to spring 1998, the prevalence of E. multilocularis in rodents was 9% to 39% for Arvicola terrestris and 10% to 21% for Microtus arvalis. From June 1996 to October 1997, 6 (7%) of 86 feral dogs and 1 of 33 cats living close to the region tested positive for intestinal E. multilocularis infection. Testing included egg detection by coproscopy, antigen detection by enzyme-linked immunosorbent assay (ELISA), and specific parasite DNA amplification by polymerase chain reaction. Thus, the presence of infected domestic carnivores can increase E. multilocularis exposure risk in humans. A seroepidemiologic survey of 2,943 blood donors in the area used specific Em2-ELISA. Comparative statistical analyses of seroprevalence and clinical incidence showed an increase in Em2-seroprevalence from 1986 and 1996-97 but no increase in clinical incidence of alveolar hydatid disease.


### Is High Prevalence of *Echinococcus multilocularis* in Wild and Domestic Animals Associated with Disease Incidence in Humans?

**Bruno Gottstein,* Francis Saucy,† Peter Deplazes,‡ Juerg Reichen,* Georges Demierre,§ Andre Busato,* Christian Zuercher,* and Paul Pugin¶**
*University of Bern, Bern, Switzerland; †University of Fribourg, Fribourg, Switzerland; ‡University of Zürich, Zürich, Switzerland; §Medecin Cantonal, Fribourg, Switzerland; and ¶Centre de Transfusion Sanguin, Hôpital Cantonal, Fribourg, Switzerland

We investigated a focus of highly endemic *Echinococcus multilocularis* infection to assess persistence of high endemicity in rural rodents, explore potential for parasite transmission to domestic carnivores, and assess (serologically) putative exposure versus infection frequency in inhabitants of the region. From spring 1993 to spring 1998, the prevalence of *E. multilocularis* in rodents was 9% to 39% for *Arvicola terrestris* and 10% to 21% for *Microtus arvalis*. From June 1996 to October 1997, 6 (7%) of 86 feral dogs and 1 of 33 cats living close to the region tested positive for intestinal *E. multilocularis* infection. Testing included egg detection by coproscopy, antigen detection by enzyme-linked immunosorbent assay (ELISA), and specific parasite DNA amplification by polymerase chain reaction. Thus, the presence of infected domestic carnivores can increase *E. multilocularis* exposure risk in humans. A seroepidemiologic survey of 2,943 blood donors in the area used specific Em2-ELISA. Comparative statistical analyses of seroprevalence and clinical incidence showed an increase in Em2-seroprevalence from 1986 and 1996-97 but no increase in clinical incidence of alveolar hydatid disease.

Alveolar hydatid disease (AHD) in humans is caused by infection with the proliferative larval stage of the small fox tapeworm *Echinococcus multilocularis*. Once the infection becomes successfully established, AHD is one of the most lethal helminthic diseases in humans (1). Infection sources, risk, and rates for humans may be related to prevalence in wild and domestic animals. In a recent study, we described highly endemic *E. multilocularis* in a small area of the canton of Fribourg, Switzerland (2). An *E. multilocularis* prevalence of 47% to 56% per year was found in the fox population. Prevalence in the local *Arvicola terrestris* population fluctuated annually between 11% and 39%. The wide distribution of *E. multilocularis* eggs in the study area, reflected by the high prevalence in rodents, may have represented a considerable risk for humans in the densely populated periurban regions of Switzerland. Recently, high *E. multilocularis* prevalence was also found in urban fox and rodent populations, which may represent an even higher infection risk for humans (3). However, in spite of high prevalence in the definitive host in most parts of Switzerland (north of the Alps), disease in humans is relatively rare. In recent decades, the annual death rate for AHD in Switzerland has been 0.18 cases per 100,000 inhabitants (4). Another study in the United States found no association between high prevalence of *E. multilocularis* in wild canids and deaths in trappers from South Dakota (5). A link between prevalence in natural definitive and intermediate wildlife hosts and domestic definitive hosts with infection risk and prevalence of disease in humans has not been examined in Switzerland.

On the basis of our previous findings (2,6,7), we designed the present study to determine the effect of a naturally occurring persistent high prevalence in wildlife hosts on domestic animals (dogs and cats) and assess (by serologic and clinical tests) the exposure rate of humans living in the area.

### Methods

The study was carried out from 1993 to 1998 in a periurban area north of the city of Fribourg. The primary (or rodent) study site has been described (2,7). A secondary area for studying the *E. multilocularis* infection rate in cats and dogs was delineated by an approximate 5-km radius around the primary site. A tertiary area for studying seroprevalence in human blood donors included approximately 20% of the surface area of the canton of Fribourg (representing approximately 400 $km^2$ surrounding the primary site).

### Survey in Intermediate Hosts (Rodents)

The rodent survey has been ongoing since spring 1993. The study period assessed in this article is spring 1993 to

Address for correspondence: B. Gottstein, Institute of Parasitology, University of Bern, Länggass Strasse 122, CH-3001 Bern, Switzerland; fax: 41-31-631-2622; e-mail: bruno.gottstein@ipa.unibe.ch spring 1998. *A. terrestris* and *Microtus arvalis* were caught as described (7) by using standardized 100-m trap lines to obtain density estimates (8). Each trapped animal was assessed for biologic variables and *E. multilocularis* infection status as described (2,7). Liver parts were preserved in 70% ethanol and 4% buffered paraformaldehyde after microscopy examination to detect metacestode lesions. All preserved lesions were subsequently assessed by immunohistochemistry or polymerase chain reaction (PCR) (2).

### Survey in Definitive Hosts (Dogs and Cats)

Three local veterinarians participated in the survey by distributing information and diagnostic fecal containers to dog and cat owners. This part of the study took place from June 1996 to October 1997. Fecal samples were examined by the following methods: Taeniid egg detection by microscopy following a flotation enrichment (9); identification of isolated *E. multilocularis* eggs by PCR (10); and detection of *E. multilocularis* antigens by sandwich-enzyme-linked immunosorbent assay (ELISA) (9). Owners of *E. multilocularis*-positive dogs or cats obtained detailed information and support by their veterinarians for treating *E. multilocularis*-infected animals and taking the necessary safety precautions, which included a recommendation to inform their physicians about previous exposure risk and to have all household members undergo serologic testing (procedures described in the next section).

### Survey in Humans

The seroepidemiologic survey was done in collaboration with the Blood Transfusion Centre, Cantonal Hospital, Fribourg. Serum samples were obtained from 2,943 blood donors in October 1996 to August 1997. The blood donation collection area included 38 villages located in the periphery of the initial rodent study area. In Switzerland, blood donation is voluntary and not compensated financially. Blood donation statistics have shown that a constant rate of 15% of the population (independent of geographic area) participates in blood donation (men four times and women three times a year). The sampling strategy was designed by the blood donation center to obtain all registered donors of the study area and avoid double donations by the same person. An identical sampling procedure and strategy had been used earlier, which also had covered approximately 15% of the study area (6). Informed consent was obtained from blood donors. All sera were stored at -30°C until tested. Em2-ELISA was performed as described, including the respective data management processing (2,6). For complementary serologic investigations, the serum samples were also tested in parallel for reactivity with an *E. granulosus* hydatid fluid antigen (EgHF) ELISA (11). If Em2-serologic test results were positive, a second blood sample was obtained through the responsible physician to rule out errors in handling the first sample and confirm the first serologic test result. If the result was confirmed, hepatic ultrasonography was performed in a regional imaging center. In cases where sonography showed hepatic lesions, the patient was referred to the University Hospital in Bern for further assessment by computed tomography (CT).

### Statistical Analyses

Comparative statistical analyses of the present seroepidemiologic data and those of a previous study (6) were done with SAS v.6.12 and Fisher's exact test (2-tail); p values were <0.005, unless otherwise stated.

### Results

*E. multilocularis* were repeatedly found in intermediate hosts (rodents). *A. terrestris* and *M. arvalis* were captured in the same period from 1993 to 1998. The presence of *E. multilocularis* metacestodes in the liver was determined by microscopy and, if required, was confirmed by immunohistochemistry and PCR. Prevalence data (Table 1) indicate that during the study period, the area maintained a relatively constant high infection rate (prevalence 9% to 39% for *A. terrestris* and 10% to 23% for *M. arvalis*). The yearly fluctuation of prevalence of *A. terrestris* was different from that of *M. arvalis*. For *A. terrestris,* a significant interannual temporal effect (p <0.005) was observed. No significant interannual effect was observed for *M. arvalis.*

### *E. multilocularis* in Definitive Hosts (Dogs and Cats)

The prevalence of intestinal *E. multilocularis* infection in dogs and cats living near the area under investigation is provided in Table 2. The definitive prevalence of 7% for dogs and 3% for cats may be underestimated as the veterinarians involved in the collection of samples informed regional cat and dog owners about the possibility of preventing infection by monthly administration of praziquantel (5 mg per kg body weight). Bias was suggested by the fact that most *E. multilocularis*-positive dogs were detected at the beginning of this study in 1996.

Table 1. Prevalence of *Echinococcus multilocularis* in *Arvicola terrestris* and *Microtus arvalis* captured in spring 1993 and 1998

| Year | No. of *A. terrestris* trapped | No. positive by microscopy/immunochemistry[a] PCR[b] | (%) | [95% CI] | No. of *M. arvalis* trapped | No. positive by microscopy/immunohistochemistry PCR | (%) | [95% CI] |
|---|---|---|---|---|---|---|---|---|
| 1993 | 28 | 11 | (39) | [21-57] | nt | -- | – | -- |
| 1994 | 44 | 5 | (11) | [ 2-20] | 20 | 2 | (10) | [ 3-23] |
| 1995 | 67 | 6 | ( 9) | [ 2-16] | 61 | 13 | (21) | [11-32] |
| 1996 | 49 | 10 | (20) | [ 9-21] | 55 | 9 | (16) | [ 7-26] |
| 1997 | 59 | 4 | ( 7) | [ 1-13] | 52 | 12 | (23) | [12-35] |
| 1998 | 46 | 4 | ( 9) | [ 1-17] | 32 | 5 | (16) | [3-28] |
| Totals | 293 | 40 | (14) | [ 1-18] | 220 | 41 | (19) | [13-24] |

[a]Positivity is based on a primary microscopy lesion detection and subsequent confirmation of *E. multilocularis* by immunohistochemistry and PCR.

[b]PCR, polymerase chain reaction; nt, not trapped.

Table 2. Prevalence of *Echinococcus multilocularis* in dogs and cats, 1996[a]

| Animal | No. of animals tested | No. positive for taeniid eggs | No. positive by PCR | No. positive by ELISA | Final *E. multilocularis* positive diagnosis (%) |
|---|---|---|---|---|---|
| Feral dogs | 86 | 7[b] | 6[bc] | 6[bc] | 6/86 (7) |
| Cats | 33 | 1 | 1 | 1 | 1/33 (3) |

[a]Polymerase chain reaction (PCR) was performed directly on eggs following a taeniid egg isolation (9).
[b]One dog had a borderline coproantigen reactivity. Subsequent investigations provided a negative *Echinococcus*-PCR by the presence of taeniid eggs. Thus, final test interpretation did not indicate *E. multilocularis* infection.
[c]PCR- and copro-Ag-positivity refers to all the same animals. The one dog exhibiting a borderline copro-Ag-reactivity is not in these group of animals.

### *E. multilocularis* in Humans

Sera from 2,943 healthy blood donors were tested by Em2-ELISA (Table 3). All six Em2-positive blood donors had a relatively high anti-Em2 antibody concentration (>15 antibody units, AU) (11). The donors were referred to their physicians for hepatic imaging analyses by ultrasonography. Four of the six donors agreed to this procedure; in three of the four, no lesions could be detected by the initial ultrasonography. The procedure was repeated approximately 6 months and 24 months after immunodiagnosis and then annually. No lesions have been detected in these three donors; one, however, had a small echodense lesion of a few centimeters in diameter in the right liver lobe (Figure, A). The donor was referred to the outpatient clinic of the University Hospital in Bern for an abdominal CT, which confirmed the presence of a small hypodense lesion assumed to be fully calcified (Figure, B). No other pathologic changes of the liver were observed. As the morphologic features of this lesion matched the criteria for nonviable, so-called “died-out” or “abortive” lesions (1,12,13), the case was classified in this category. Further CT investigations using contrast enhancement detected a very small hypodense area in the periphery of the calcified herd, which could have harbored a putatively still viable parasitic zone (Figure, C). The patient was reexamined by CT at the same time intervals as described above for the other seropositive donors. No changes in size and morphologic features of the calcified lesion and the peripheral hypodense zone have been demonstrated. The patient received no treatment at any time. The inert lesion was definitively rated as abortive in 1999.

Table 3. Specific seroprevalences for *Echinococcus multilocularis* in blood donors[a]

| | No. | (%) | [95%-CI] |
|---|---|---|---|
| Blood donors tested (total) | 2,943 | | |
| Em2-positive blood donors[b] | 6[b] | (0.2) | [0.04-0.36] |
| EgHF-positive blood donors but negative by Em2 | 33[c] | (1.1) | [0.5-2.1] |

[a]Assessed primarily by Em2-enzyme-linked immunosorbent assay (ELISA), complementary serologic data were obtained with the *E. granulosus* hydatid fluid antigen-ELISA. Blood samples were collected from October 1996 to July 1997.
[b]Four of six Em2-positive blood donors received imaging investigations, one of which was computed tomography-positive for an alveolar hydatid disease (AHD) lesion. Two Em2-positive donors refused subsequent imaging investigations but have had no signs of AHD.
[c]Ten donors with high *E. granulosus* hydatid fluid antigen-titers but negative in Em2-ELISA were selected for pilot imaging investigations. One had a cystic hepatic lesion of 2.5 cm in diameter. Morphologic features were compatible with those of *E. granulosus* cysts.

In parallel, all sera were serologically tested by EgHF-ELISA to compare data with those from a former study performed in Switzerland in 1985-86 (2). Of 2,943 sera, 33 were seropositive in this EgHF ELISA (including the six Em2-positive sera described above). Ten EgHF ELISA-positive (but Em2-negative) persons were arbitrarily selected for a subsequent hepatic ultrasonography investigation. Nine persons had negative ultrasonography results. One person, however, had a typical hepatic *E. granulosus* hydatid cyst of 5 cm in diameter and was referred to his physician for further clinical testing and treatment.

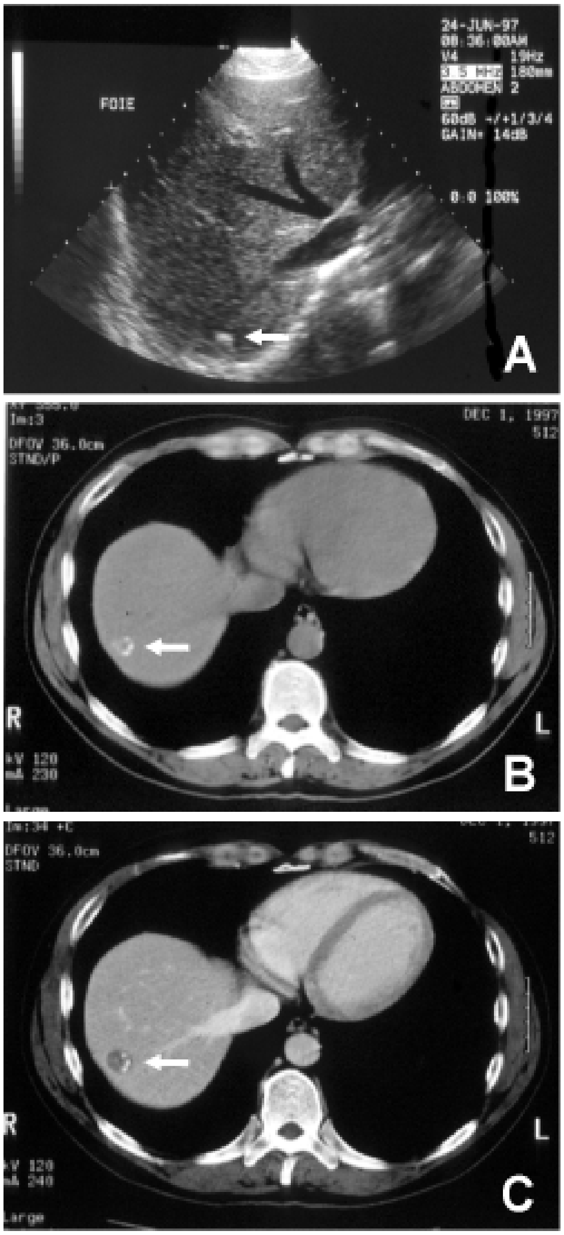

Figure. Abortive alveolar echinococcosis detected in a 44-year-old blood donor in Switzerland. A, ultrasonography of the liver demonstrates the presence of a small echodense lesion (arrow). B, a liver computed tomography scan shows a small, hypodense, apparently fully calcified lesion (arrow). C, after contrast enhancement, a very small hypodense area in the periphery of the calcified herd was detected (arrow).

### Retrospective Statistical Comparative Analyses

Seroprevalence and clinical findings obtained in the present study (Table 3) were directly compared with those obtained in a previous study, which had used similar immunodiagnostic tools and blood donor population (6). The key data used from this previous study originated from 17,166 blood donors, six Em2-ELISA-positive (two of these with hepatic lesions by CT). Subsequent surgery confirmed the presence of active *E. multilocularis* metacestodes. Of 5,166 blood donors additionally tested with EgHF-ELISA, 16 Em2-ELISA-negative donors had EgHF seropositivity. Echinococcosis tests and standard laboratory tools were the same in both studies (11): Diagnostic sensitivity of the Em2-ELISA was 95%, and specificity was 100%. Diagnostic sensitivity of the EgHF-ELISA was 96%, and specificity was 97%. By these key data and the 2-tailed Fisher's exact test, a significant increase of seroprevalence from 1986 to 1996-97 became apparent for both the Em2-ELISA ($p < 0.005$) and the EgHF-ELISA ($p < 0.01$). Conversely, clinical findings (CT hepatic lesions compatible with *E. multilocularis* infection) were not significantly different between the two studies ($p = 0.378$).

### Conclusion

Recent findings have clearly documented a persistently high prevalence of *E. multilocularis* in rural and urban fox populations of Switzerland (3,9,11,14). These data contrasted to a persistently low annual incidence of AHD (8 to 10 new cases per year) in accidentally infected humans (6,15,16). Until 1996, little information was available about the prevalence of *E. multilocularis* in the intermediate rodent host. In 1996 (2), we reported a focus of high *E. multilocularis* prevalence not only in foxes (annual mean 51%) but also in rodents (annual mean 25%). The latter represented the highest prevalence ever reported for rodents in central Europe. The discovery and documentation of an area highly endemic for *E. multilocularis* in definitive and intermediate hosts raised the following question: Does high endemicity have implications for the rate of infection in domestic animals living near *E. multilocularis*-endemic areas and for human health? To address this question, we first had to demonstrate persistence of high endemicity in the rodent population. A 6-year follow-up showed that—independent of interannual fluctuations significant for *A. terrestris* but not for *M. arvalis*—both species remained infected at an exceptionally high level. Consequently, the local dog and cat populations hunting rodents were at a persistently high infection risk. This risk may result in a relatively high exposure risk for human populations in the vicinity. The number of dogs and cats in Switzerland is relatively high (approximately 500,000 dogs and 1.2 million cats). Local veterinarians assessed the dog and cat populations of our study area as within the Swiss average (1 dog per 10 inhabitants and 1 cat per 5 inhabitants). Therefore, any person in our study area may have had direct contact with pet dogs or cats or may have been to locations contaminated by their feces. The veterinarians confirmed that dog and cat owners in the study area did not exhibit any peculiarities in comparison to owners in other areas of Switzerland, including the area and period covered by an earlier study (6). As a precaution, all pet owners visiting veterinary practices in the study area were given information on *E. multilocularis*. The information indicated that carnivores eating rodents in this specific area could be prophylactically treated every 28 days with a therapeutic dose (5 mg per kg body weight) of praziquantel. Assessment of the effectiveness of this therapeutic regimen will be the topic of a separate study.

When we compared our study to other European studies (17,18), we confirmed (as a consequence of the persistently high prevalence in intermediate rodent hosts) an exceptionally high prevalence of intestinal *E. multilocularis* infections, especially in dogs. High prevalence among foxes, dogs, cats, and rodents reflects high environmental contamination with *E. multilocularis* eggs, putatively brought into the households by dogs and cats. Thus, we examined the extended exposure risk of the local human population for increases in seroprevalence, by comparing current data with those collected in an earlier study (6). As expected, Em2-ELISA exhibited a significantly higher seroprevalence. (Higher seroprevalence obtained with the EgHF-ELISA will not be further discussed because the lower specificity of the test may be due to other nonspecific parameters.) However, the number of reported clinical cases did not increase. This lack of increase in cases was underlined by the unique detection of one abortive (died-out) case of AHD (first such case documented in Switzerland). Earlier, we had postulated that the time between infection and clinical manifestation was 5 to 15 years (4,6). However, we know that, in experimental infections of rodents, seroconversion to the Em2-antigen occurs as early as 4 to 6 weeks after peroral inoculation of *E. multilocularis* eggs (unpub. data). As our study covered a 4- to 5-year period of high endemicity (1993 to 1996-97) until the human population was assessed, a significant increase in clinical cases, including asymptomatic (early) cases, which are detectable by ultrasonography, should have been observed in our study. However, high prevalence of *E. multilocularis* in wild (and domestic animals) seemed not to be associated with a higher prevalence of AHD in humans living in the same region.

Epidemiologic data similar to ours have recently been reported with regard to a rural community in southwestern Germany where a high prevalence of *E. multilocularis* (75%) had been observed in foxes (13). Screening of the human population (2,560 participants) found one case of active AHD and nine cases of seropositivity to specific antibodies without detectable liver lesions.

The human population in our study area exhibited low susceptibility to AHD: The relatively high seroprevalence observed in the population was associated with the documented high exposure rate, but the disease rate did not increase. (Disease cases included early cases putatively detectable by ultrasonography.) In addition to low susceptibility, the persistently low incidence of AHD in our study area may also be accounted for by increased immunity, which may protect a large proportion of infected persons. For more detailed and definitive conclusions on disease incidence in humans, we will continue to monitor the affected population. A long-term assessment of the same human population is already planned in the Fribourg area over the next 4 to 10 years. By using the same tools as described in this project, it will be possible to document more subtle changes in disease prevalence in humans.

### Acknowledgments

We thank A. Renz, Y. Tingeuely, and M. Berther for excellent veterinary support; S. Moirandaz, C. Repond, and J.O. Maillard for assisting with blood donations; G. Ammann, F. Detraz, J. Skaggs, and E. Felleisen for organizing blood donations, laboratory samples, and questionnaires; and A. Hemphill for helpful suggestions and critical comments on the manuscript.

The study was supported by the Swiss National Science Foundation (Project No. 31-45575.95) and the Bundesamt fur Wirtschaft and Arbeit (Interreg II; No. 30.027).

### References

1. Gottstein B, Felleisen R. Protective immune mechanisms against the metacestode of *Echinococcus multilocularis.* Parasitol Today 1995;11:320-6.
2. Gottstein B, Saucy F, Wyss C, Siegenthaler M, Jacquier P, Schmitt M, et al. Investigations on a Swiss area highly endemic for *Echinococcus multilocularis.* Appl Parasitol 1996;37:129-36.
3. Hofer S, Gloor S, Müller U, Mathis A, Hegglin D, Deplazes P. High prevalence of *Echinococcus multilocularis* in urban red foxes (*Vulpes vulpes*) and voles (*Arvicola terrestris*) in the city of Zürich, Switzerland. Parasitology 2000;120:135-42.
4. Ammann RW, Hoffmann AF, Eckert J. Swiss study of chemotherapy of alveolar echinococcosis—review of a 20-year clinical research project. Schweiz Med Wochenschr 1999;129:323-32.
5. Hildreth MB, Sriram S, Gottstein B, Wilson M, Schantz PM. Failure to identify alveolar echinococcosis in trappers from South Dakota in spite of high prevalence of *Echinococcus multilocularis* in wild canids. J Parasitol 2000;86:75-7.
6. Gottstein B, Lengeler C, Bachmann P, Hagemann P, Kocher P, Brossard M, et al. Sero-epidemiological survey for alveolar echinococcosis (by Em2-ELISA) of blood donors in an endemic area of Switzerland. Trans R Soc Trop Med Hyg 1987;81:960-4.
7. Schmitt M, Saucy F, Wyborn S, Gottstein B. Befall von Schermäusen (*Arvicola terrestris*) mit Metazestoden von *Echinococcus multilocularis* im Kanton Freiburg (Schweiz). Schweiz Arch Tierheilkd 1997;139:84-93.
8. Pascal M, Meylan A. L'échanillonage linéaire des populations de la forme fouisseuse du Campagnol terrestre (*Arvicola terrestris scherman* (Shaw)). Défense des Végétaux 1986;225:36-44.
9. Deplazes P, Alther P, Tanner I, Thompson RC, Eckert J. *Echinococcus multilocularis* coproantigen detection by enzyme-linked immunosorbent assay in fox, dog, and cat populations. J Parasitol 1999;85:115-21.
10. Mathis A, Deplazes P, Eckert J. An improved test system for PCR-based specific detection of *Echinococcus multilocularis* eggs. J Helminthol 1996;7:219-22.
11. Gottstein B, Jacquier P, Bresson-Hadni S, Eckert J. Improved primary immunodiagnosis of alveolar echinococcosis in humans by an enzyme-linked immunosorbent assay using the Em2plus-antigen. J Clin Microbiol 1993;31:373-6.
12. Rausch RL, Wilson JF, Schantz PM, McMahon BJ. Spontaneous death of *Echinococcus multilocularis*: cases diagnosed serologically by Em2-ELISA and clinical significance. Am J Trop Med Hyg 1987;36:576-85.
13. Romig T, Kratzer W, Kimmig P, Frosch M, Gaus W, Flegel WA, et al. An epidemiological survey of human alveolar echinococcosis in southwestern Germany. Am J Trop Med Hyg 1999;6:566-73.
14. Ewald D, Eckert J, Gottstein B, Straub M, Nigg H. Parasitological and serological studies on the prevalence of *Echinococcus multilocularis* Leuckart, 1863 in red foxes (*Vulpes vulpes* Linnaeus, 1758) in Switzerland. Rev Sci Techn 1992;11:1057-61.
15. Ammann RW, Ilitsch N, Marincek B, Freiburghaus AU. Effect of chemotherapy on the larval mass and the long-term course of alveolar echinococcosis. Swiss Echinococcosis Study Group. Hepatol 1994;19:735-42.
16. Eckert J, Deplazes P. Alveolar echinococcosis in humans: the current situation in Central Europe and the need for countermeasures. Parasitol Today 1999;15:315-9.
17. Viel JF, Giraudoux P, Abrial V, Bresson-Hadni S. Water vole (*Arvicola terrestris* scherman) density as risk factor for human alveolar echinococcosis. Am J Trop Med Hyg 1999;61:559-65.
18. Petavy AF, Deblock S, Walbaum S. Life cycles of *Echinococcus multilocularis* in relation to human infection. J Parasitol 1991;77:133-7.
